# Intrasellar Tuberculosis Presenting as Pituitary Apoplexy: A Case Report

**DOI:** 10.7759/cureus.75378

**Published:** 2024-12-09

**Authors:** Shankar Ayyappan Kutty, Naemieh Mohammad Kamel Aljasem, Anagha Shankar, Sagar Shahane

**Affiliations:** 1 Neurosciences, NMC Specialty Hospital, Abu Dhabi, ARE; 2 Department of Pathology, NMC Royal Hospital, Abu Dhabi, ARE; 3 Anatomical Sciences, Humanitas University, Milan, ITA; 4 Department of Neurosurgery, NMC Royal Hospital, Abu Dhabi, ARE

**Keywords:** adenoma, apoplexy, atypical sellar lesion, pituitary gland, sellar mass, sellar-parasellar lesion, sellar tumor, tuberculosis

## Abstract

Patients presenting with acute onset of headache and ophthalmoplegia are clinically diagnosed as having a pituitary adenoma with apoplexy. Rarely, other diseases can mimic this condition clinically and radiologically, requiring a high index of suspicion to reach the correct diagnosis. We present a case of a 37-year-old male of Indian origin, who had intra- and supra-sellar tuberculosis (TB), presenting with classical clinical features of pituitary apoplexy and constitutional symptoms. Following surgery, he was started on anti-tuberculous therapy, and his condition improved over the next few months. Intrasellar tuberculoma should be considered among the differential diagnoses, especially in persons coming from areas endemic for TB, and in immunocompromised patients.

## Introduction

Tuberculosis (TB) involving the central nervous system is rare, accounting for about 1% of all cases of TB reported worldwide, with most cases being reported from endemic areas such as the Indian subcontinent. Primary pituitary TB is an even rarer disorder with only about a hundred cases reported worldwide [1], and presentation with an apoplexy is extremely rare, with only five cases reported previously [2,3]. We report the first such case report from the United Arab Emirates. The patient was a diabetic Indian male, who presented with features suggestive of a pituitary apoplexy. The report outlines clinical findings and management and also discusses the challenges in the diagnosis and management of these cases.

## Case presentation

A 37-year-old Indian male presented to the internal medicine outpatient clinic with a history of acute onset of headache and multiple episodes of vomiting in June 2023. He had one episode of loss of consciousness on the same day and had been suffering from diplopia in all directions of gaze since then. Clinical examination showed third, fourth, and sixth nerve paresis on the right side. Fundus examination did not reveal any papilledema. Visual field charting was not reliable as the patient was not cooperative for the testing. On admission, he was detected to have uncontrolled diabetes mellitus, which was managed with insulin. He also had a fever of unknown origin, with negative urine and blood cultures, and he was started empirically on antibiotics for the same. There was no history of TB either for himself or in the immediate family.

**Figure 1:**
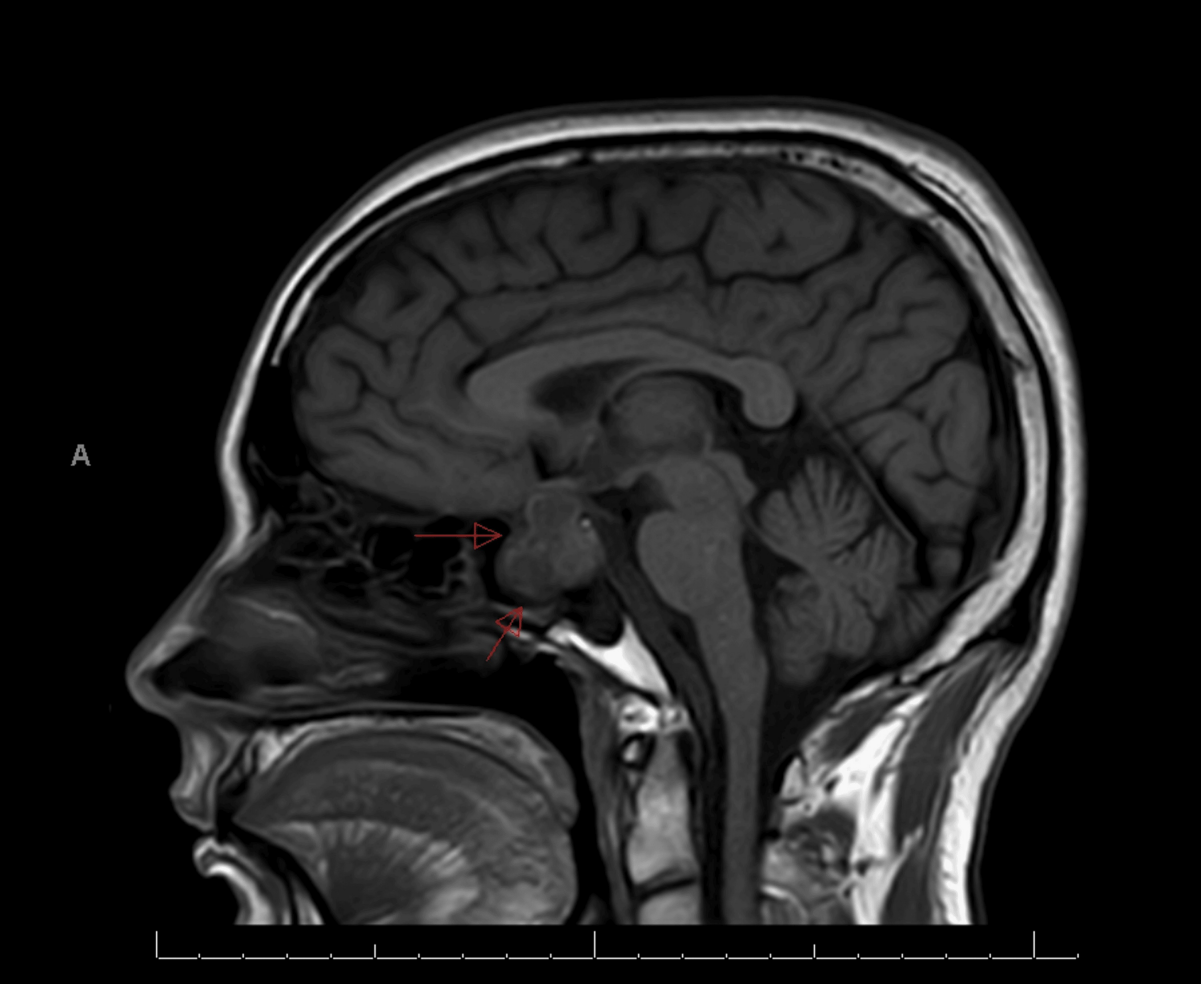
Preoperative magnetic resonance imaging T1-weighted (MRI T1W) sagittal view The red arrows point to the sellar-suprasellar tumor, which has a slightly heterogenous intensity.

**Figure 2:**
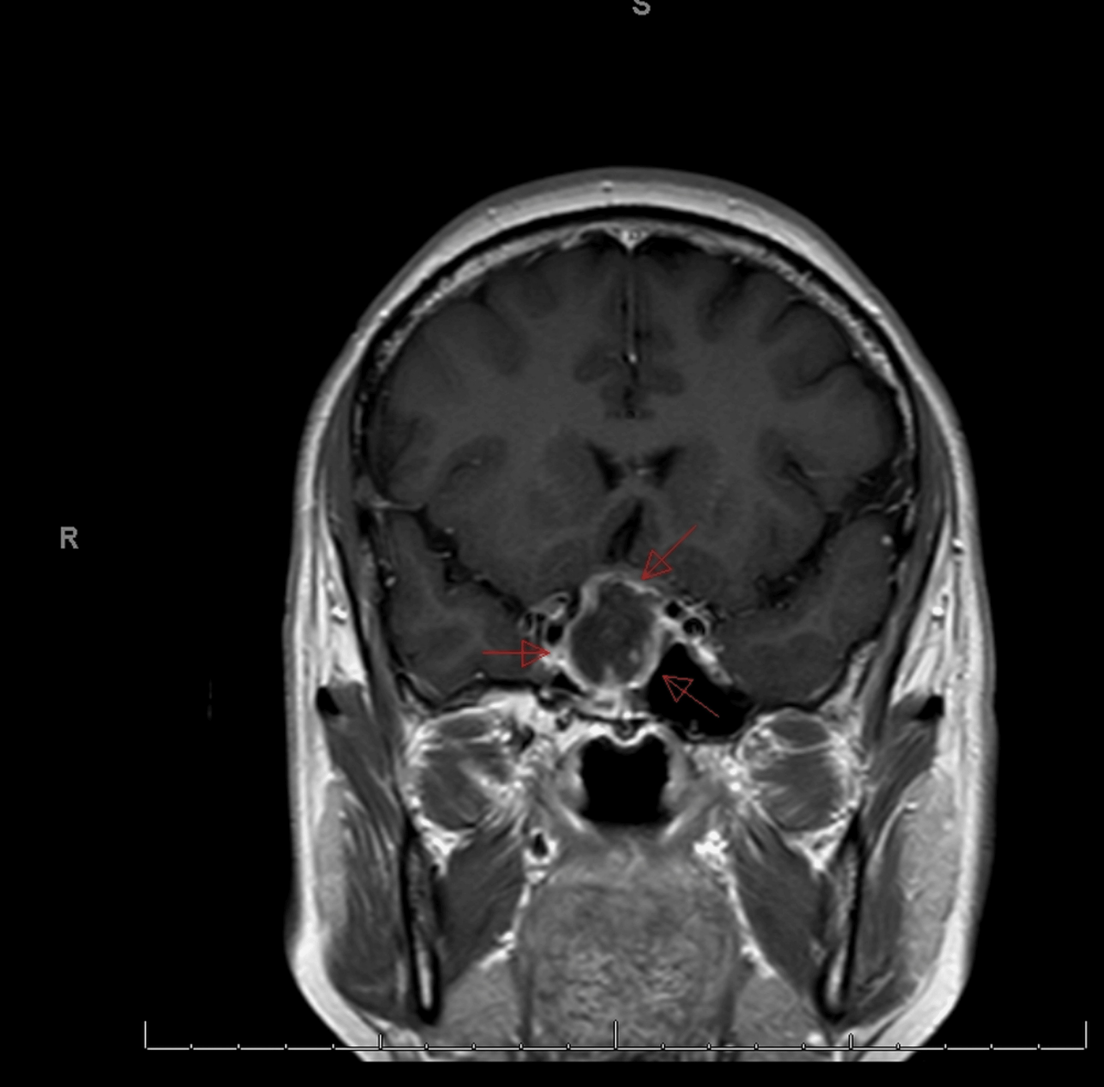
Contrast-enhanced T1-weighted (T1W) coronal magnetic resonance imaging (MRI) The peripheral enhancement of the lesion, which is eccentric to the right side, is well visualized in the coronal sequences.

Magnetic resonance imaging (MRI) study of the brain with contrast showed a large sellar-suprasellar lesion, 3.2 x 2.2 x 2.5cm in size. It was heterogenously isointense to the gray matter on both T1- and T2-weighted images. No evidence of restricted diffusion was present. There was mild peripheral enhancement and few linear areas of internal enhancement with a mostly non-enhancing center on the postcontrast scan. The lesion involved the right cavernous sinus with encasement of the internal carotid artery on that side. The optic chiasm could not be visualized separately from the lesion. The lesion was extending into the sphenoid sinus on its right side, with erosion of the sellar floor (Figures 1-2). Following the MRI, a diagnosis of pituitary macroadenoma with apoplexy was made, and he was transferred under Neurosurgery for further management.

The preoperative hormonal assay showed low cortisol levels. Other pituitary hormones were within normal ranges. An endocrinology consultation was done, and he was started on parenteral hydrocortisone, following which he was taken up for urgent surgery. 

Intraoperatively, the sellar floor was found to be deficient on the right side, where the tumor was bulging into the sphenoid sinus. The tumor was yellowish-white in color, relatively avascular, firm, and not amenable to suction or curettage. It was densely adherent to the overlying dura and the arachnoid on top of the sella. No cyst was encountered. The solid component was adherent to, and infiltrating, the cavernous sinus on both sides and had to be partly left behind since there was severe bleeding from the sinus on trying to dissect the lesion off the sinus wall and there was a risk of injury to the carotid artery while trying to dissect an adherent and firm tumor from its walls. There was a small tear in the arachnoid on the left side, with a cerebrospinal fluid (CSF) leak, and the arachnoid was seen pouching into the sella at the end of the procedure.

**Figure 3:**
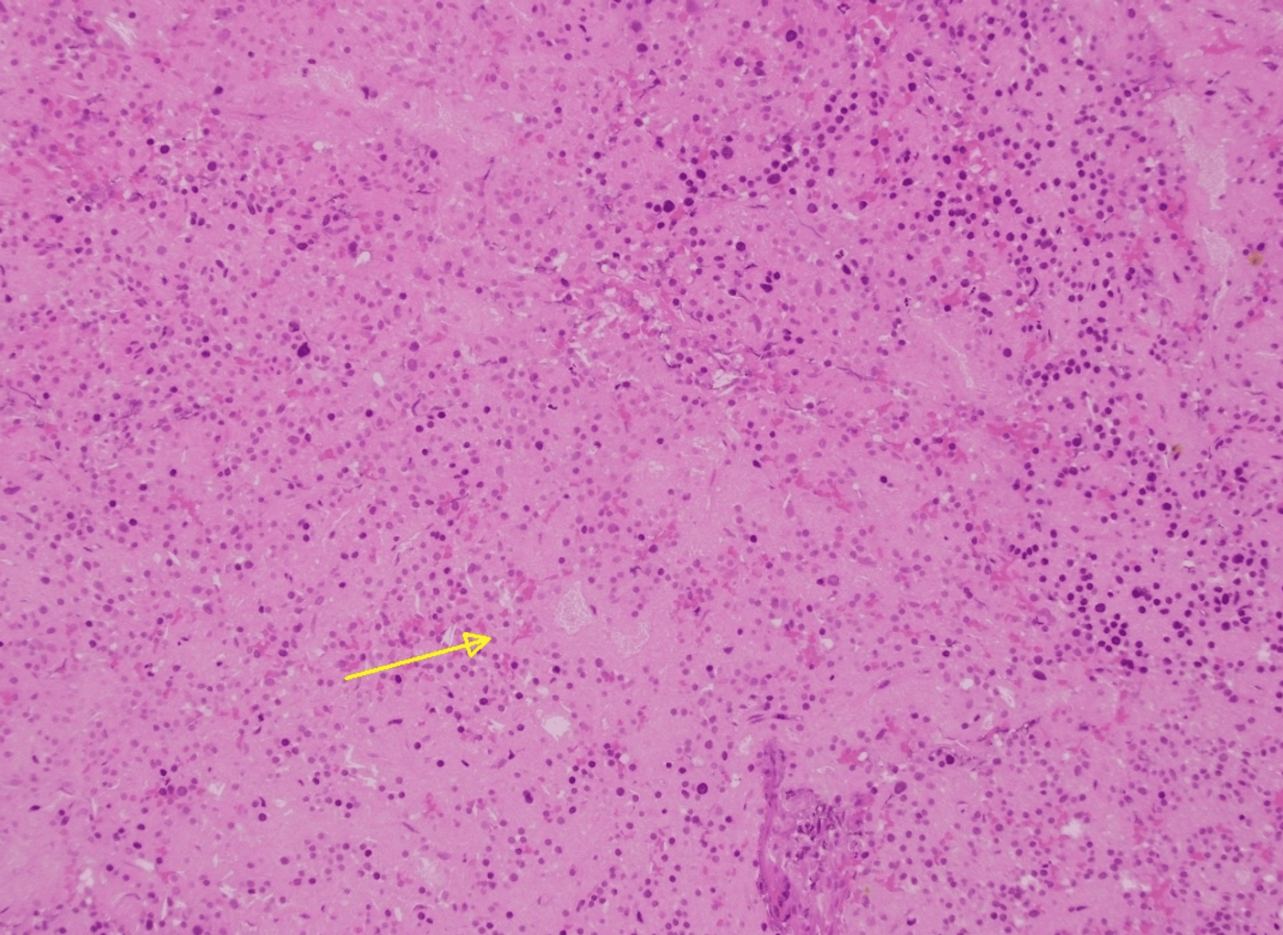
Hematoxylin and eosin stain at x200 magnification showing a central area of necrosis

**Figure 4:**
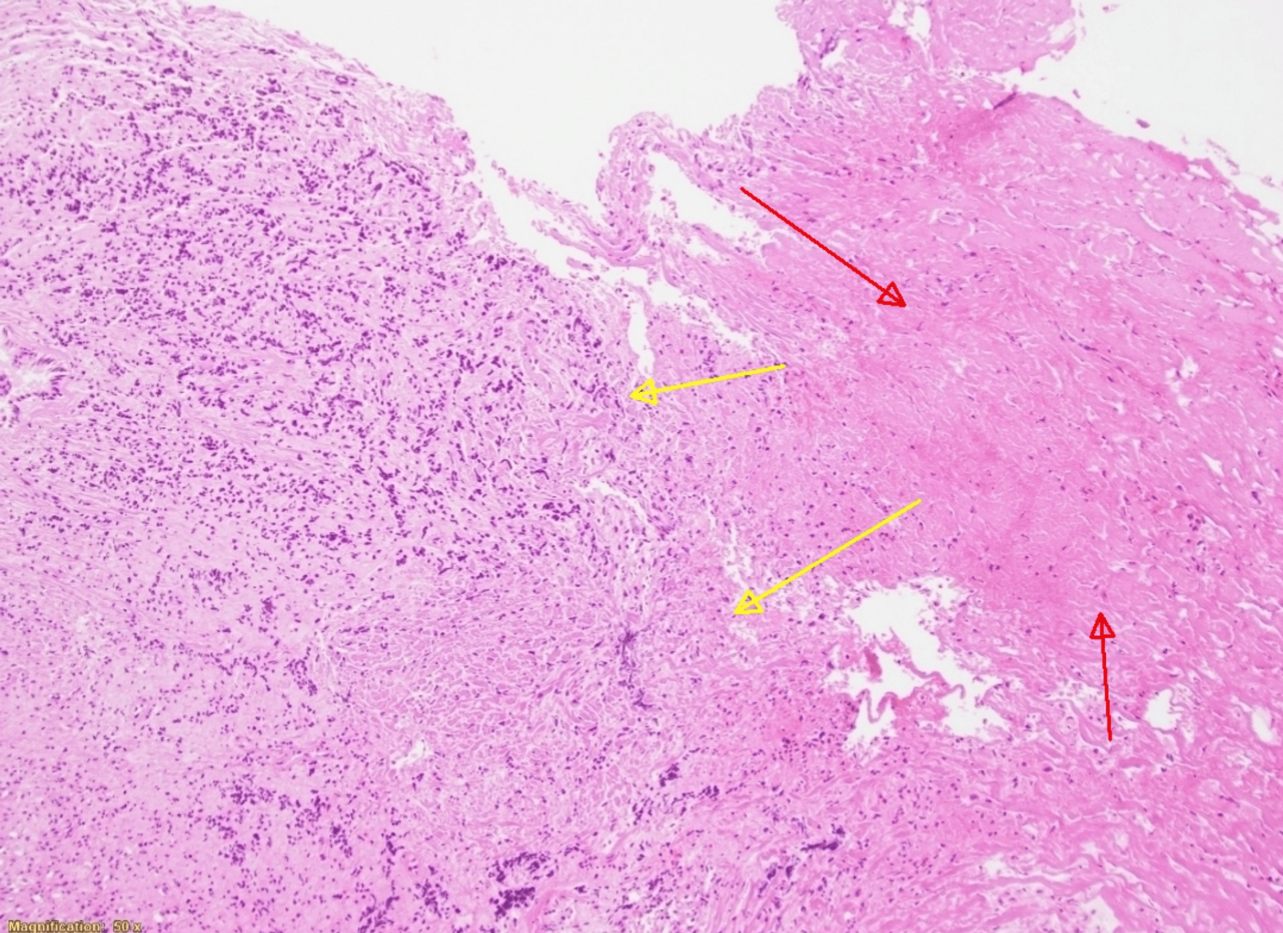
Hematoxylin and eosin stain at x100 magnification showing normal pituitary gland (yellow arrows) with adjacent area of necrosis (red arrows)

Histopathology revealed a wall of a cavity containing a central core of necrotic debris/tissue and collections of neutrophils (Figure 3), surrounded by a variably developed rim of granulation tissue with mixed inflammatory infiltrates and maturing blood vessels, accompanied by foci of the pituitary gland (Figure 4).

**Figure 5:**
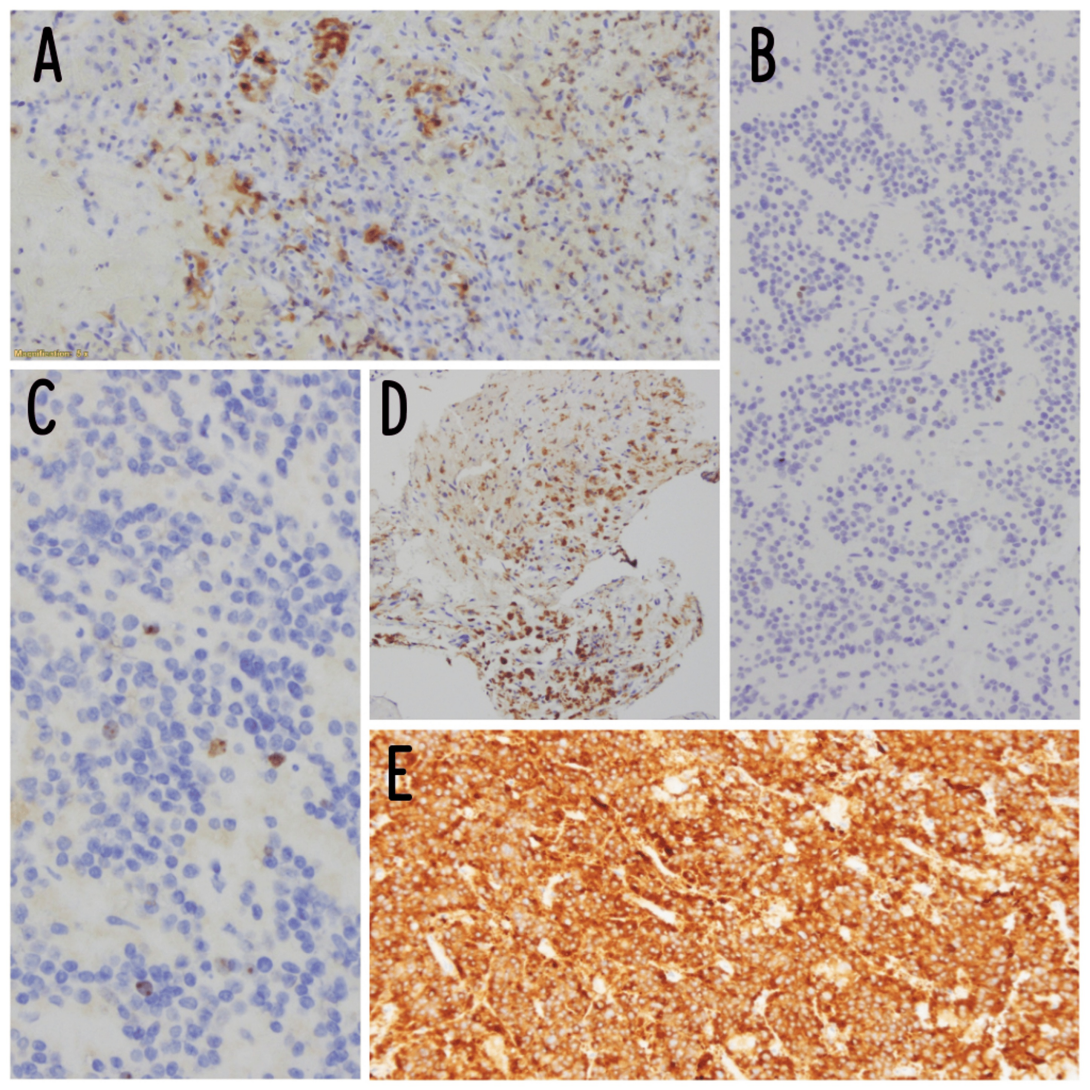
Immunohistochemistry images of the tumor A: S100, B: p53, C: ki67, D: CD68, and E: synaptophysin

Immunohistochemistry results are provided in Table 1, and the corresponding images can be seen in Figure 5.

**Table 1:** Immunohistochemistry of the tumor

Immunohistochemistry	Result
S100 (Polyclonal)	Positive for scattered cells
P53 (DO-7)	Negative
Ki-67 (MIB-1)	<2%
CD68 (514H12)	Focal positive
Synaptophysin (DAK-SYNAP)	Diffuse positive at viable and necrotic gland

**Figure 6:**
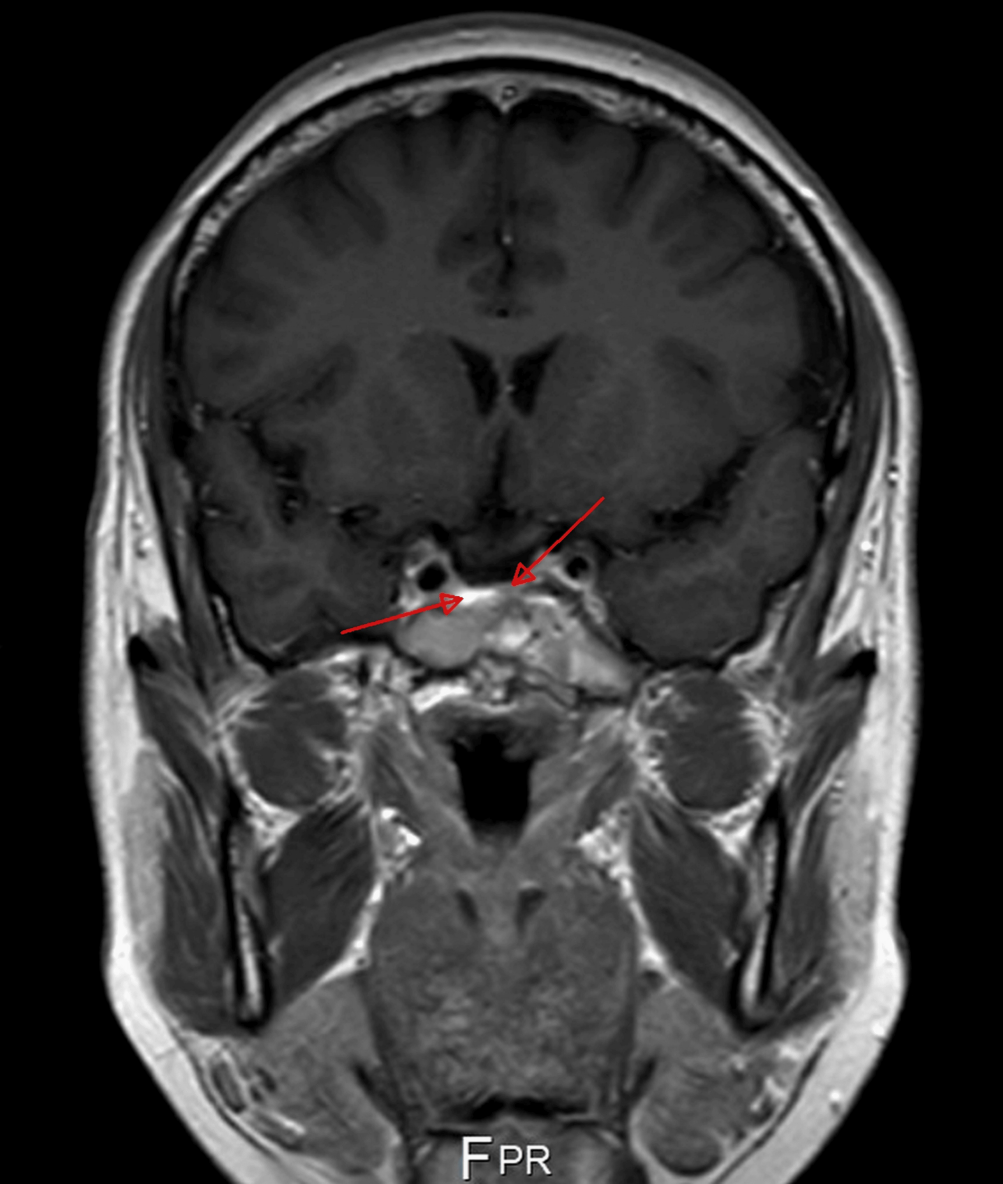
MRI with contrast showing resolution of the lesion six months after the surgery

Tissue staining for acid-fast bacilli and fungus was negative. Cultures from the tissue were sent for aerobic organisms, mycobacteria, and fungi, and all these were reported to be negative. QuantiFERON-TB Gold+ was also negative. Considering the histopathological evidence for a granulomatous abscess, and the history of fever at the time of presentation, he was started on a four-drug anti-tuberculous regimen comprising isoniazid (INH), rifampicin, ethambutol, and pyrazinamide. After two months, ethambutol and pyrazinamide were discontinued as per the protocol of the Department of Health, Abu Dhabi, United Arab Emirates. Three months after surgery, his gaze paresis had resolved and he had no symptoms. The hormonal profile had become normal, and oral steroids were discontinued by the endocrinologist. A follow-up MRI scan six months later showed no residual intrasellar tuberculoma (Figure 6).

## Discussion

TB remains one of the top 10 causes of death worldwide, with an increase in the number of people falling ill with drug-resistant TB being reported in 2022 [3,4]. It is becoming more common in developed countries because of co-infections with human immunodeficiency virus (HIV), multidrug-resistant bacteria, and an increase in travel and migration; therefore, pituitary TB is seen more frequently [5].

Pituitary tuberculomas account for 0.15-4% of all intracranial masses. Sharma et al. reported a series of 18 pituitary tuberculomas in which all but one mimicked a pituitary adenoma both clinically and radiologically, and diagnosis was made on histological examination alone [6]. As of 2021, approximately 106 cases of pituitary tuberculomas have been reported, of which 51 were from India. The male-to-female ratio was 1:2.55 (male: 29, female: 74 out of 103) [7]. Over the last two decades, there has been a slight reduction in the average age of patients presenting with pituitary tuberculomas, with a mean age of 35.41 in 2021 compared to 36.7 in 2001 [7,8]. A review of existing literature by Srisukh et al. in 2016 found only four cases of pituitary tuberculoma presenting with apoplexy, including their own publication [3,8,9,10]. A fifth case was reported by Krishnan et al. in 2024 [2], and our case would be the sixth such instance to be reported (Table 2).

**Table 2:** Published cases of pituitary tuberculosis presenting with apoplexy-like picture. All patients who underwent surgery were noted to have firm tumors, not amenable to suction. All the patients improved with anti-tuberculous medication, but the duration is not mentioned in all reports. The range is from nine to 18 months.

Case report	Patient profile	Clinical presentation	Hormonal profile	Radiology findings	Histopathology
Arunkumar MJ et al., 2001 [8]	27-year-old male	Three episodes of headache, the last being one month prior to the hospital visit, associated with altered sensorium and visual blurring, which improved gradually	Normal	CT scan: Hemorrhage within the pituitary lesion, MRI scan six weeks later showed a reduction in the size of the lesion with a thick stalk.	Small fragments of fibrotic, hyalinised connective tissue. Focal aggregates of lymphocytes. Ill-defined granulomata composed of epitheloid histiocytes.
Deogaonkar M et al., 2005 [9]	27-year-old female	Sudden onset of headache, ptosis of the left eye, numbness in the left V1-V2 dermatomes, Glasgow Coma Scale score = E3 M6 V4	Slight elevation in prolactin	CT scan: Enhancing sellar and suprasellar mass with evidence of hemorrhage. MRI scan: Heterogeneous enhancement of mass and pituitary stalk	Dense aggregates of lymphoid cells, occasional plasma cells, a few ill-defined granulomas composed of epitheloid cells and Langhans-type multinucleated giant cells.
Verma R et al., 2014 [10]	17-year-old female	Sudden onset headache and vomiting for 10 days followed by severe bilateral visual loss and altered sensorium. Glasgow Coma Scale score = E2 M4 V2	Elevated TSH, otherwise within normal limits	MRI scan: Multiple ring-enhancing lesions in the sella and suprasellar region with meningeal enhancement and hydrocephalus	No surgery. Cerebrospinal fluid and tuberculosis polymerase chain reaction (TB-PCR) were positive.
Srisukh S et al., 2016 [3]	25-year-old female	Headache, rapidly progressive visual loss in both eyes for five days	Pan hypopituitarism	MRI scan: Rim-enhancing lesion	Necrotizing granulomatous inflammation with multinucleated giant cells and infarction of the pituitary parenchyma
Krishnan G et al., 2024 [2]	21-year-old female, diabetic	Lethargy and vomiting for three days, treated as diabetic ketoacidosis (DKA). Sudden loss of vision in both eyes and severe headache three days after admission.	Hypothyroidism and hypogonadism	MRI scan: Sellar-suprasellar lesion with erosion into the sphenoid sinus. Post-contrast rim enhancement noted.	Only necrotic tissue. Immunohistochemistry testing of the tissue using GeneXpert MTB/Rif was positive with indeterminate rifampicin resistance, which suggested a tuberculoma.
Ayyppan Kutty S et al. (this case)	37-year-old male, diabetic	Headache, vomiting, fever, and diplopia one day	Hypocortisolism	MRI scan: Sellar-suprasellar lesion with peripheral enhancement	The central core of necrotic debris and collections of neutrophils, surrounded by a rim of granulation tissue

Common clinical symptoms are headaches, visual disturbances, low-grade fever, and vomiting [7]. Thus, our patient had a classical presentation of a pituitary tuberculoma. Although headache was a symptom present in all patients with intrasellar tuberculoma, there were only a few cases where there was a severe headache suggestive of apoplexy or subarachnoid hemorrhage [11,12].

A diagnosis of tuberculoma or sarcoidosis should be suspected in patients with sellar-suprasellar tumors, especially those who present with a history suggestive of apoplexy when the lesion is found to be firm and not amenable to suction during surgery. Since the risk of injury to the carotid artery is very high while attempting to dissect these firm lesions, it is more prudent to perform a biopsy and plan further treatment based on the histopathological diagnosis. The preferred surgical route is a transsphenoidal approach, as it can allow for tumor decompression and tissue diagnosis without cerebrospinal fluid contamination [13].

In all of the cases described by Ranjan and Chandy [14], the abnormal tissue was grayish or yellowish, firm, moderately vascular to avascular, and relatively non-suckable, similar to the findings in our case. The sellar floor is usually minimally thinned out, and the dura matter may appear thickened during surgery.

A review of 52 cases of pituitary TB with a positive pathological diagnosis showed that acid-fast bacilli were identified in the sample or grown on culture in less than 10% of the cases. Only one case was reported to have a positive QuantiFERON TB-Gold interferon-gamma release assay. As such, it is usually necessary to start anti-tuberculous medications empirically once granulomatous lesions have been identified on histopathology [10].

Anti-tuberculous treatment for nine to 24 months is recommended, depending on the clinical and imaging outcome [7]. Our patient had no symptoms three months after surgery, and his anti-tuberculous treatment was discontinued after nine months.

As pituitary adenomas constitute only about 85% of the lesions that occur in the sellar region, physicians and surgeons should be aware of the various other lesions that may occur in the pituitary fossa. The second commonest lesion reported in this region is craniopharyngioma, followed by cystic non-neoplastic lesions such as Rathke’s cleft cysts. Inflammatory diseases such as fungal granulomas, TB, sarcoidosis, and hypophysitis are next, and various neoplasms that range from benign to malignant may also present in the sellar-suprasellar area. Meningiomas constitute about 0.94% of tumors in the sella, while chordomas, sarcomas, lymphomas, and metastases have been rarely reported [15,16].

## Conclusions

TB involving the pituitary gland is rare, but it can sometimes even present with symptoms suggestive of an apoplexy. It is important to consider this diagnosis when the intraoperative findings are not consistent with an adenoma, as there is no need to attempt a radical excision of these lesions, especially since such attempts may result in inadvertent injury to the internal carotid arteries. In addition, appropriate investigations such as staining and culture for TB can be ordered at the time of surgery only if a clinical suspicion exists.
